# Dependence of Initial Value on Pattern Formation for a Logistic Coupled Map Lattice

**DOI:** 10.1371/journal.pone.0158591

**Published:** 2016-07-06

**Authors:** Li Xu, Guang Zhang, Haoyue Cui

**Affiliations:** School of Science, Tianjin University of Commerce, Tianjin, China; Shanxi University, CHINA

## Abstract

The logistic coupled map lattices (LCML) have been widely investigated as well as their pattern dynamics. The patterns formation may depend on not only fluctuations of system parameters, but variation of the initial conditions. However, the mathematical discussion is quite few for the effect of initial values so far. The present paper is concerned with the pattern formation for a two-dimensional Logistic coupled map lattice, where any initial value can be linear expressed by corresponding eigenvectors, and patterns formation can be determined by selecting the corresponding eigenvectors. A set of simulations are conducted whose results demonstrate the fact. The method utilized in the present paper could be applied to other discrete systems as well.

## Introduction

Logistic coupled map lattices (LCML) are important models to investigate spatially extended dynamical systems recognized as such since the early 80s. Logistic coupled map lattices present discrete space and time, but a continuous state variable whose evolution is governed by a map. Thus, Logistic coupled map lattices are able to generate local information and a rich spatio-temporal dynamics. Such properties encourage the use of LCMLs as models to describe the behavior of chemical and biological systems, magnetic and optical media, gas and electron hole plasmas, semiconductor and gas-discharge structures, etc [1].

LCML can be defined as cases of coupling which takes into account the effects of the nearest neighbors of a given lattice site, that can be viewed as a discretization of a second order spatial derivative appearing in a diffusive term of reaction–diffusion systems. A two-dimensional LCML can be defined as

$$u_{ij}^{t+1}=\left(1-\varepsilon \right)f\left(u_{ij}^{t}\right)+\frac{\varepsilon }{4}\left[f\left(u_{i+1,j}^{t}\right)+f\left(u_{i,j+1}^{t}\right)+f\left(u_{i-1,j}^{t}\right)+f\left(u_{i,j-1}^{t}\right)\right]$$ (1)

where

$$\nabla^{2}f\left(u_{ij}^{t}\right)=f\left(u_{i+1,j}^{t}\right)+f\left(u_{i,j+1}^{t}\right)+f\left(u_{i-1,j}^{t}\right)+f\left(u_{i,j-1}^{t}\right)-4f\left(u_{ij}^{t}\right).$$ (2)

and *ε* is the coupling parameter, the mapping function *f*(*x*) = *λx*(1 − *x*), and *λ* ∈ (0, 4].

The two-dimensional logistic coupled map lattice is exploited to describe the Turing instability in [2]. The different pattern structures have also been observed for same parameters and different initial values. Indeed, an example has been shown for *ε* = 0.34 and *λ* = 2.9 in [2]. We find that different patterns such as stripe pattern and spiral pattern resulting from random initial values will emerge, even if all parameters are fixed. It is an interesting fact.

Patterns formation may depend on both fluctuations of system parameters and variation of the initial conditions. Due to a large number od degrees of freedom, a rich variety of spatio-temporal solutions is available for those spatial systems in different regions of parameter space. As the system parameters are changed, the qualitative structure of solutions for certain parameters will vary [3–4]. So far, most theoretical and experimental investigations for continuous or discrete reaction-diffusion systems have focused on the parameters of systems, for example, see [5–15]. The initial concentration also plays a great role for system evolvement, such as population evolution, genetic program and chemical reaction. Initial distribution of an immobile reactive species can affect pattern formation [16–23]. For example, comparison of spatio–temporal evolution of experimental subaqueous particulate gravity flows at two different initial concentrations is made in [16]. The extent of malic acid degradation is affected by its initial concentration, the extent and the rate of deacidification increased with initial malate concentration [17]. In the absence of an electric field pattern formation exhibits increasingly stochastic behavior as the initial concentration difference between the outer and the inner electrolytes (D) approaches to zero [18]. Average host density per cell (equivalent to metapopulation density), plotted against time, illustrate how metapopulation behavior and spatial patterns can differ depending on initial conditions, even when parameter values are identical [19]. [20] shows that the convergence to periodic attractors and the sensitivity of chaotic processes of the logistic map depends not only on the control parameter but also on the eigenvalues of the matrix of initial conditions.

From different viewpoint, We will prove that the asymptotic behavior of the problem Eqs (1) and (2) depend on the eigenvalues and corresponding eigenvectors of a discrete Laplace operator. That is, any initial value can be linear expressed by eigenvectors, and we can obtain different pattern by means of the selective eigenvectors. It will have theoretical significance for pattern dynamics.

The remainder of this paper is organized as follows. Firstly, we will show how the different initial distributions have differential effect on the spatiotemporal dynamics of the two-dimensional logistic coupled map lattice. Secondly, numerical results will show that different patterns can be realized by means of selecting different eigenvector as initial value in stable or unstable space. Finally, we summarize our results.

## Methods

### Eigenvalue Analysis

In this section, we will assume that *ε* > 0 is the coupling parameter, *m* is a positive integer, *i*, *j* ∈ {1, 2, …, *m*} = [1, *m*], the mapping function *f*(*x*) = *λx*(1 − *x*), and *λ* ∈ (1, 3). In this case, the system (1) can be rewritten by

$$u_{ij}^{t+1}=f\left(u_{ij}^{t}\right)+\varepsilon \nabla^{2}f\left(u_{ij}^{t}\right),$$ (3)

where

$$\nabla^{2}f\left(u_{ij}^{t}\right)=f\left(u_{i+1,j}^{t}\right)+f\left(u_{i,j+1}^{t}\right)+f\left(u_{i-1,j}^{t}\right)+f\left(u_{i,j-1}^{t}\right)-4f\left(u_{ij}^{t}\right).$$ (4)

For the sake of convenience, we denote $\frac{\varepsilon }{4}$ by *ε* in Eq (4) yet. It is well known that the linearization equation of Eq (3) can be written by

$$u_{ij}^{t+1}=\left(2-\lambda \right)\left(u_{ij}^{t}+\varepsilon \nabla^{2}u_{ij}^{t}\right).$$ (5)

To clearly illustrate our views, we also need to consider eigenvalues of the following equation

$$\nabla^{2}X^{ij}+\lambda X^{ij}=0\;$$ (6)

with the periodic boundary conditions

$$X^{i,0}=X^{i,m},X^{i,1}=X^{i,m+1}$$ (7)

and

$$X^{0,j}=X^{m,j},X^{1,j}=X^{m+1,j}.$$ (8)

In view of [2], the eigenvalue problem Eqs (6)–(8) has the eigenvalues

$$\lambda_{ls}=4\left(\sin^{2}\frac{(l-1)\pi }{m}+\sin^{2}\frac{(s-1)\pi }{m}\right)=k_{ls}^{2}$$ (9)

and the corresponding eigenvectors

$$v_{ij1}^{ls}=\sin\frac{2(l-1)\pi i}{m}\sin\frac{2(s-1)\pi j}{m}$$ (10)

$$v_{ij2}^{ls}=\sin\frac{2(l-1)\pi i}{m}\cos\frac{2(s-1)\pi j}{m}$$ (11)

$$v_{ij3}^{ls}=\cos\frac{2(l-1)\pi i}{m}\sin\frac{2(s-1)\pi j}{m}$$ (12)

$$v_{ij4}^{ls}=\cos\frac{2(l-1)\pi i}{m}\cos\frac{2(s-1)\pi j}{m}forl,s\in \left[1,m\right].$$ (13)

From [2], we easily see that the conditions of Turing instability for the problem Eqs (1) and (2) are: there exist *l*, *s* ∈ [1, *m*] and *ε* > 0 such that

$$\varepsilon >\frac{\lambda -1}{4\left(\lambda -2\right)\left(\sin^{2}\frac{(l-1)\pi }{m}+\sin^{2}\frac{(s-1)\pi }{m}\right)}\;\text{for}\;2<\lambda <3$$ (14)

or

$$\varepsilon >\frac{3-\lambda }{4\left(2-\lambda \right)\left(\sin^{2}\frac{(l-1)\pi }{m}+\sin^{2}\frac{(s-1)\pi }{m}\right)}\;\text{for}\;1<\lambda <2.$$ (15)

### Dependence of Initial Value

Now, let $u_{ij}^{0}=v_{ij}^{ls}$, then we have

$$u_{ij}^{1}=\left(2-\lambda \right)\left(v_{ij}^{ls}+\varepsilon \nabla^{2}v_{ij}^{ls}\right)$$ (16)

$$=\left(2-\lambda \right)\left(v_{ij}^{ls}-\varepsilon \lambda_{ls}v_{ij}^{ls}\right)$$ (17)

$$=\left(2-\lambda \right)\left(1-\varepsilon \lambda_{ls}\right)v_{ij}^{ls},$$ (18)

$$u_{ij}^{2}\;=\;\left(2-\lambda \right)\left[\left(2-\lambda \right)\left(1-\varepsilon \lambda_{ls}\right)v_{ij}^{ls}+\varepsilon \left(2-\lambda \right)\left(1-\varepsilon \lambda_{ls}\right)\nabla^{2}v_{ij}^{ls}\right]$$ (19)

$$=\left(2-\lambda \right)\left(2-\lambda \right)\left(1-\varepsilon \lambda_{ls}\right)\left(1-\varepsilon \lambda_{ls}\right)v_{ij}^{ls}$$ (20)

$$={\left(2-\lambda \right)}^{2}{\left(1-\varepsilon \lambda_{ls}\right)}^{2}v_{ij}^{ls},$$ (21)

… (22)

$$u_{ij}^{t}={\left[\left(2-\lambda \right)\left(1-\varepsilon \lambda_{ls}\right)\right]}^{t}v_{ij}^{ls}.$$ (23)

For any initial value function $u_{ij}^{0}$, it can be expressed by

$$u_{ij}^{0}=\sum_{l,s=1}^{m}c_{ls}v_{ij}^{ls}.$$ (24)

Thus, we have

$$u_{ij}^{t}={\left(2-\lambda \right)}^{t}\left[\sum_{l,s=1}^{m}c_{ls}{\left(1-\varepsilon \lambda_{ls}\right)}^{t}v_{ij}^{ls}\right]$$ (25)

$$={\left(2-\lambda \right)}^{t}\left[\sum_{l,s=1}^{m}{\left(-1\right)}^{t}c_{ls}{\left(\varepsilon \lambda_{ls}-1\right)}^{t}v_{ij}^{ls}\right].$$ (26)

Let *λ*_*LS*_ = max_*l*,*s*_{*λ*_*ls*_} and assume that *c*_*LS*_ ≠ 0, then, we have

$$\frac{u_{ij}^{t}}{{\left[\left(2-\lambda \right)\left(\varepsilon \lambda_{LS}-1\right)\right]}^{t}}=\sum_{l,s=1}^{m}{\left(-1\right)}^{t}c_{ls}\frac{{\left(\varepsilon \lambda_{ls}-1\right)}^{t}}{{\left(\varepsilon \lambda_{LS}-1\right)}^{t}}v_{ij}^{ls}$$ (27)

$$\to c_{LS}v_{ij}^{LS}.$$ (28)

From above discussion, we find that the solution ${\left\{u_{ij}^{t}\right\}}_{i,j\in \left[1,m\right]}^{t\in Z^{+}}$ of Eq (5) and the sequence

$${\left\{c_{LS}{\left[\left(2-\lambda \right)\left(\varepsilon \lambda_{LS}-1\right)\right]}^{t}v_{ij}^{LS}\right\}}_{i,j\in \left[1,m\right]}^{t\in Z^{+}}$$ (29)

have some asymptotic behavior.

In the following, we study the sequence

$${\left\{{\left[\left(2-\lambda \right)\left(\varepsilon \lambda_{LS}-1\right)\right]}^{t}v_{ij}^{LS}\right\}}_{i,j\in \left[1,m\right]}^{t\in Z^{+}}.$$ (30)

First of all, we assume that 1 < *λ* < 2. In this case, we have

$$\left(2-\lambda \right)\left(\varepsilon \lambda_{LS}-1\right)>1,$$ (31)

which implies that the sequence {[(2 − *λ*)(*ελ*_*LS*_ − 1)]^*t*^}_*t* ∈ *Z*^+^_ is monotone increased and

$$\lim_{t\to \infty }{\left[\left(2-\lambda \right)\left(\varepsilon \lambda_{LS}-1\right)\right]}^{t}=+\infty .$$ (32)

Thus, we think that the “good” patterns cannot be observed. If 2 < *λ* < 3, we have

$$\left(\lambda -2\right)\left(\varepsilon \lambda_{LS}-1\right)>1,$$ (33)

the sequence {[(2 − *λ*)(*ελ*_*LS*_ − 1)]^*t*^}_*t* ∈ *Z*^+^_ is oscillation.

## Results

### Stable and Unstable Space

Corresponding to the above theory analysis, the initial value $u_{ij}^{0}$ is chosen by

$$u_{ij}^{0}=\frac{\lambda -1}{\lambda }+\delta \sum_{l,s=1}^{m}c_{ls}v_{ls}^{ij},$$ (34)

here *δ* is small enough. From Eq (5), we know that the number of eigenvalues for the eigenvalue problem Eqs (6)–(8) is *m*^2^, where $k_{11}^{2}=0$ is a unique simple eigenvalue. According to Section 2, numerical simulations will be given for different *m*.

For some fixed parameters, we denote unstable space

$$E^{u}=span\left\{v_{ij}^{ls}\left|\left|\lambda_{ls}\right|>1,\left(l,s\right)\in {\left[1,m\right]}^{2}\right.\right\}$$ (35)

and stable space

$$E^{s}=span\left\{v_{ij}^{ls}\;\left|\left|\lambda_{ls}\right|<1,\left(l,s\right)\in {\left[1,m\right]}^{2}\right.\right\}.$$ (36)

When a initial value $u_{ij}^{0}$ is chosen, clearly, some of *c*_*ls*_ may be zero or $\sum_{l,s=1}^{m}c_{ls}v_{ls}^{ij}\in E^{s}$, then we have naturally

$$u_{ij}^{t}\to \frac{\lambda -1}{\lambda }\;\text{as}\;t\to \infty .$$ (37)

If there exists *c*_*ls*_ ≠ 0 or $\sum_{l,s=1}^{m}\;c_{ls}v_{ls}^{ij}\in E^{u}$, the solution of Eq (3) will be away from the equilibrium.

### Numerical Simulation

In the following, we will perform a series of numerical simulations of the two-dimensional Logistic coupled map lattice in two-dimensional spaces. When *m* is even, first of all, we shall show some dynamics of the system if *c*_*ls*_ = 0 or $\sum_{l,s=1}^{m}\;c_{ls}v_{ls}^{ij}\in E^{s},$ only stable pattern can be observed.

When there exist *c*_*ls*_ ≠ 0 for $\sum_{l,s=1}^{m}\;c_{ls}v_{ls}^{ij}\in E^{u}$, we firstly consider patterns if

$$\varepsilon >\frac{3-\lambda }{4\left(2-\lambda \right)\left(\sin^{2}\frac{(l-1)\pi }{m}+\sin^{2}\frac{(s-1)\pi }{m}\right)}\;\text{for}\;1<\lambda <2.$$ (38)

**Fig 1:**
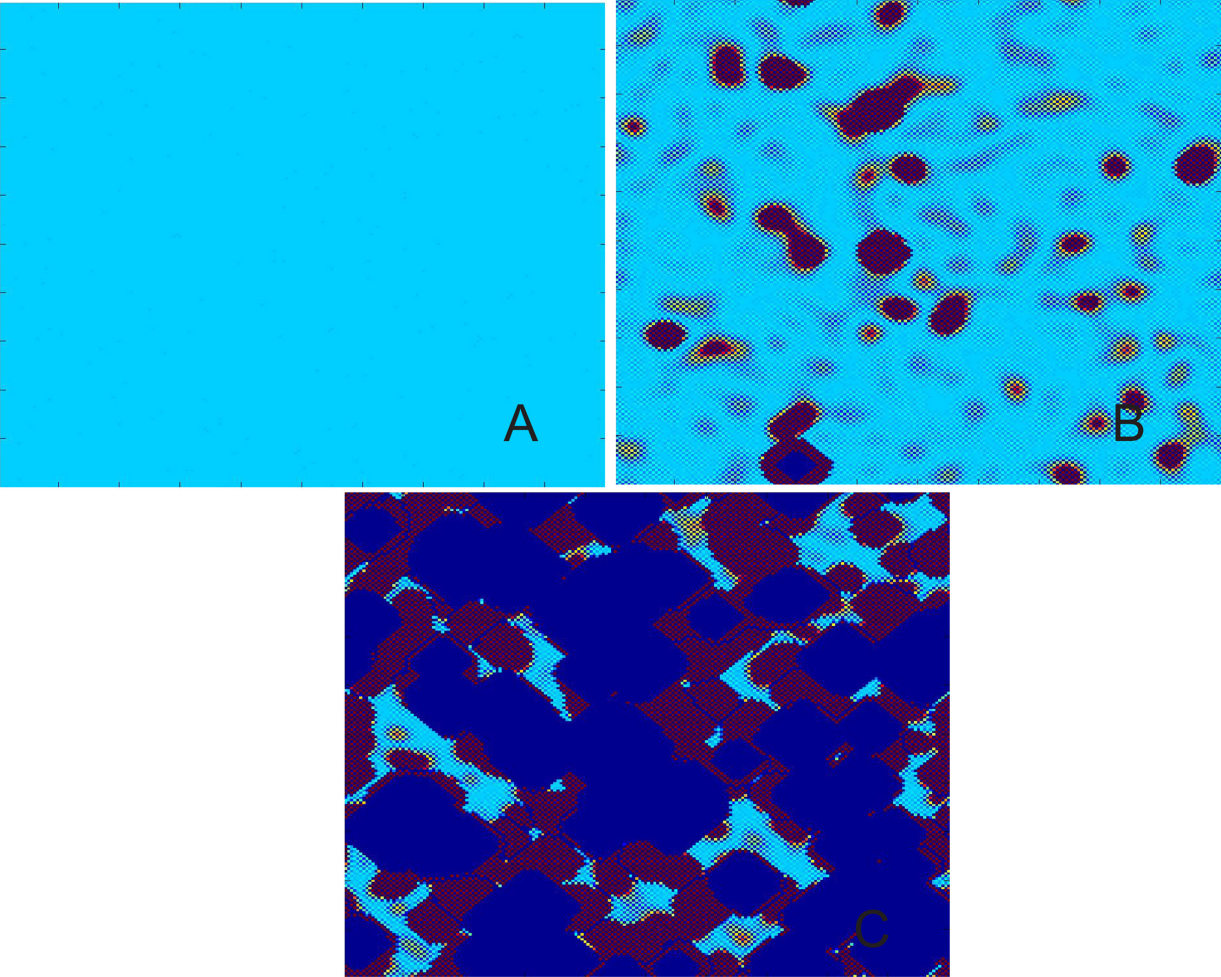
Spatial pattern of the time evolution at different instants. Snapshots of contour pictures of the time evolution of CML system at some instants with *λ* = 1.5 and *ε* = 0.4 in the Turing instability region. (A) *t* = 0. (B) *t* = 57. (C) *t* = 69.

Fig 1 shows snapshots of transient pattern at 0, 57, and 69 iterations for the parameter *λ* = 1.5 and *ε* = 0.4 with a system size of 200 × 200 space units. If the iteration is further increased, the boundary of the domain moves in time till a single domain covers the space which we call ‘not good’ pattern or ‘overflowing’ pattern. Even if other parameters in the above parameter space are selected, similar fact will be observed, which only has different time to a single domain.

Next, we assume that the condition

$$\varepsilon >\frac{\lambda -1}{4\left(\lambda -2\right)\left(\sin^{2}\frac{(l-1)\pi }{m}+\sin^{2}\frac{(s-1)\pi }{m}\right)}\;\text{for}\;2<\lambda <3$$ (39)

hold. To explore clearly if different initial distributions have differential effect on the spatiotemporal dynamics of the two dimensional coupled map lattices, we investigate the effect of initial value by keeping the system parameters of the system fixed. As a numerical example, a series of simulations firstly are finished with a system size of 200 × 200 space units when *λ* = 2.9 and *ε* = 0.34.

**Fig 2:**
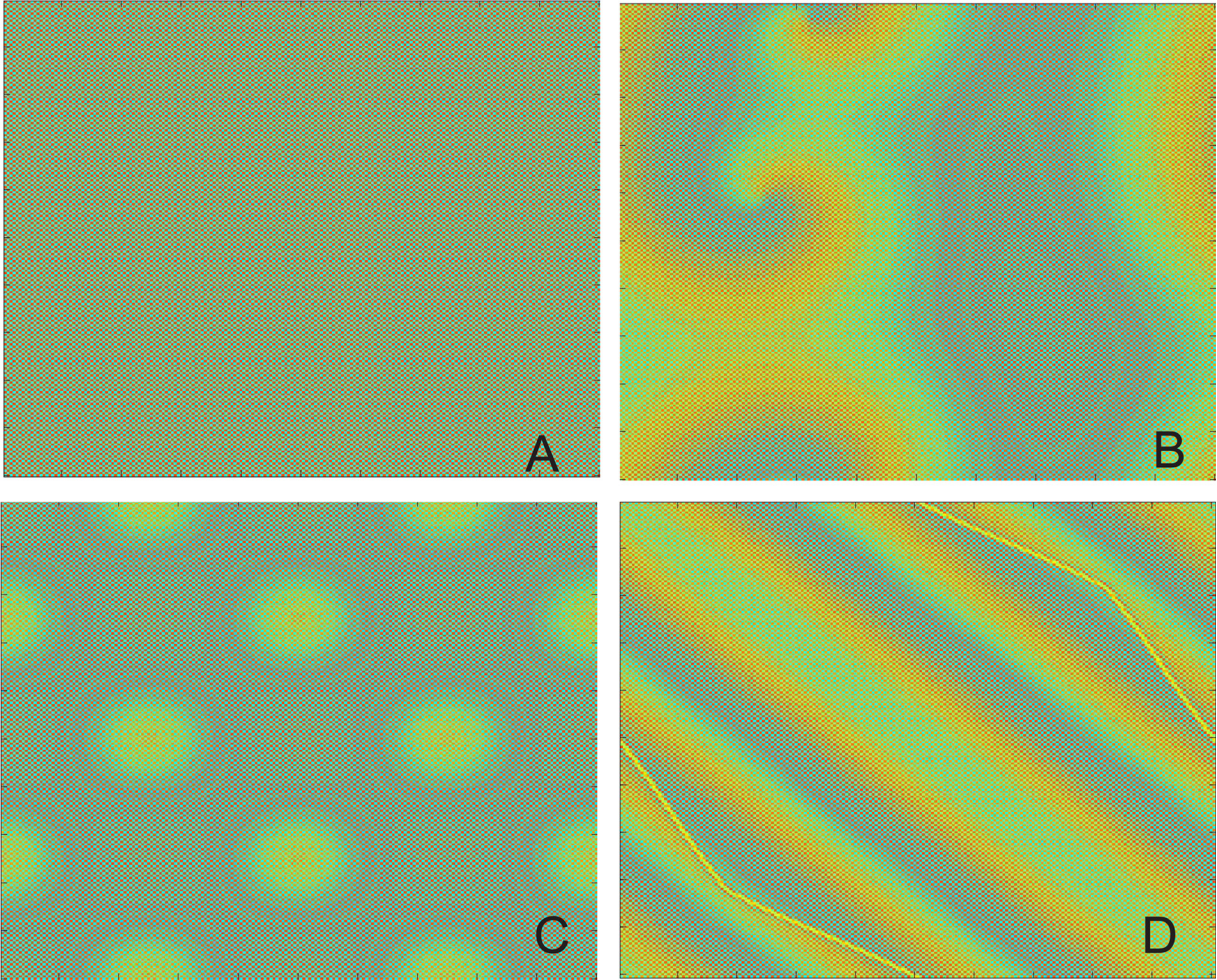
Spatial pattern at given times with different initial value $\left(\left(\lambda -1\right)/\lambda \right)+\delta v_{ij}^{ls}$. Snapshots of the spatial grid at given times *t* for various *l*, *s* when the initial value is selected as $\left(\left(\lambda -1\right)/\lambda \right)+\delta v_{ij}^{ls}$ with *δ* = 0.01, *λ* = 2.9 and *ε* = 0.34. (A) *l* = 2, *s* = 2, *t* = 50000. (B) *l* = 5, *s* = 50, *t* = 50000. (C) *l* = 50, *s* = 50, *t* = 5000. (D) *l* = 5, *s* = 95, *t* = 50000.

**Fig 3:**
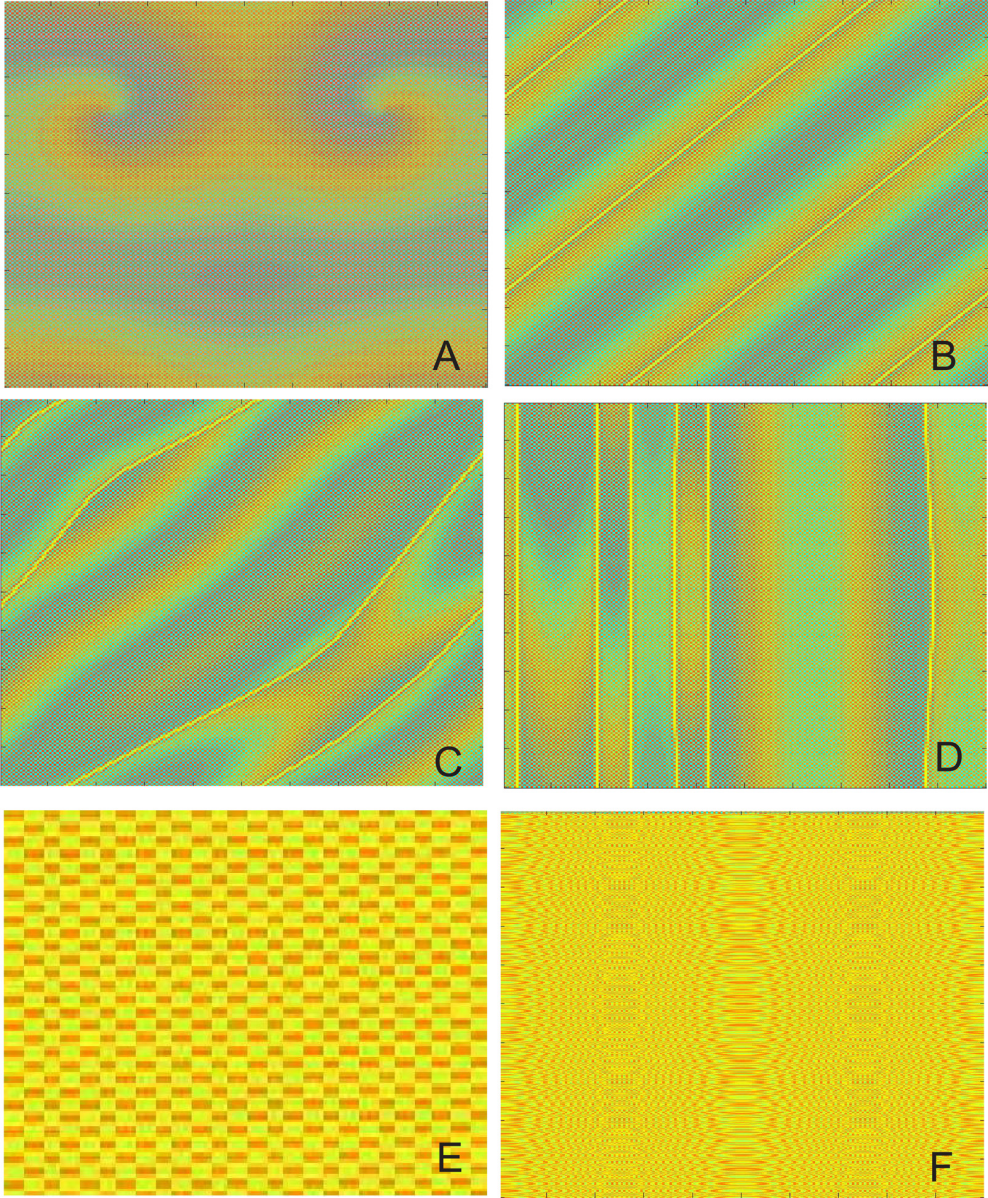
Spatial pattern at given times with different initial value $\left(\left(\lambda -1\right)/\lambda \right)+\delta \sum_{l,s}^{}\delta_{ls}v_{ij}^{ls}$. Snapshots of the spatial grid at given times *t* for various *l*, *s* when the initial value is selected as $\left(\left(\lambda -1\right)/\lambda \right)+\delta \sum_{l,s}^{}\delta_{ls}v_{ij}^{ls}$ with *δ* = 0.01, *λ* = 2.9 and *ε* = 0.34. (A) spiral wave. (B) traveling wave with the diagonal direction of spatial propagation. (C) trigger wave with the diagonal direction of spatial propagation. (D) trigger wave with the vertical direction of spatial propagation. (E) stationary wave. (F) spatiotemporal chaos.

Fig 2 shows some snapshots of the spatial grid at given times *t* for various *l*, *s* when the initial value is selected as $\frac{\lambda -1}{\lambda }+\delta v_{ij}^{ls}$. Let *δ* = 0.01, as shown in Fig 2A For *l* = 2, *s* = 2, a stable pattern of square shapes, namely, stationary wave is observed. But if we let *l* = 5, *s* = 50, spiral patterns will emerge in Fig 2B. When *l* = 5 and *s* = 95, clear stripe patterns appear in Fig 2C. An interesting situation is depicted in Fig 2D where a transient dot-like pattern can be seen when *l* = 50 and *s* = 50. If the initial distributions are further changed, similar patterns are observed. Moreover, for different initial value like $\frac{\lambda -1}{\lambda }+\delta \sum_{l,s}^{}\delta_{ls}v_{ij}^{ls}$ or some special initial values, various patterns can be seen in Fig 3.

**Fig 4:**
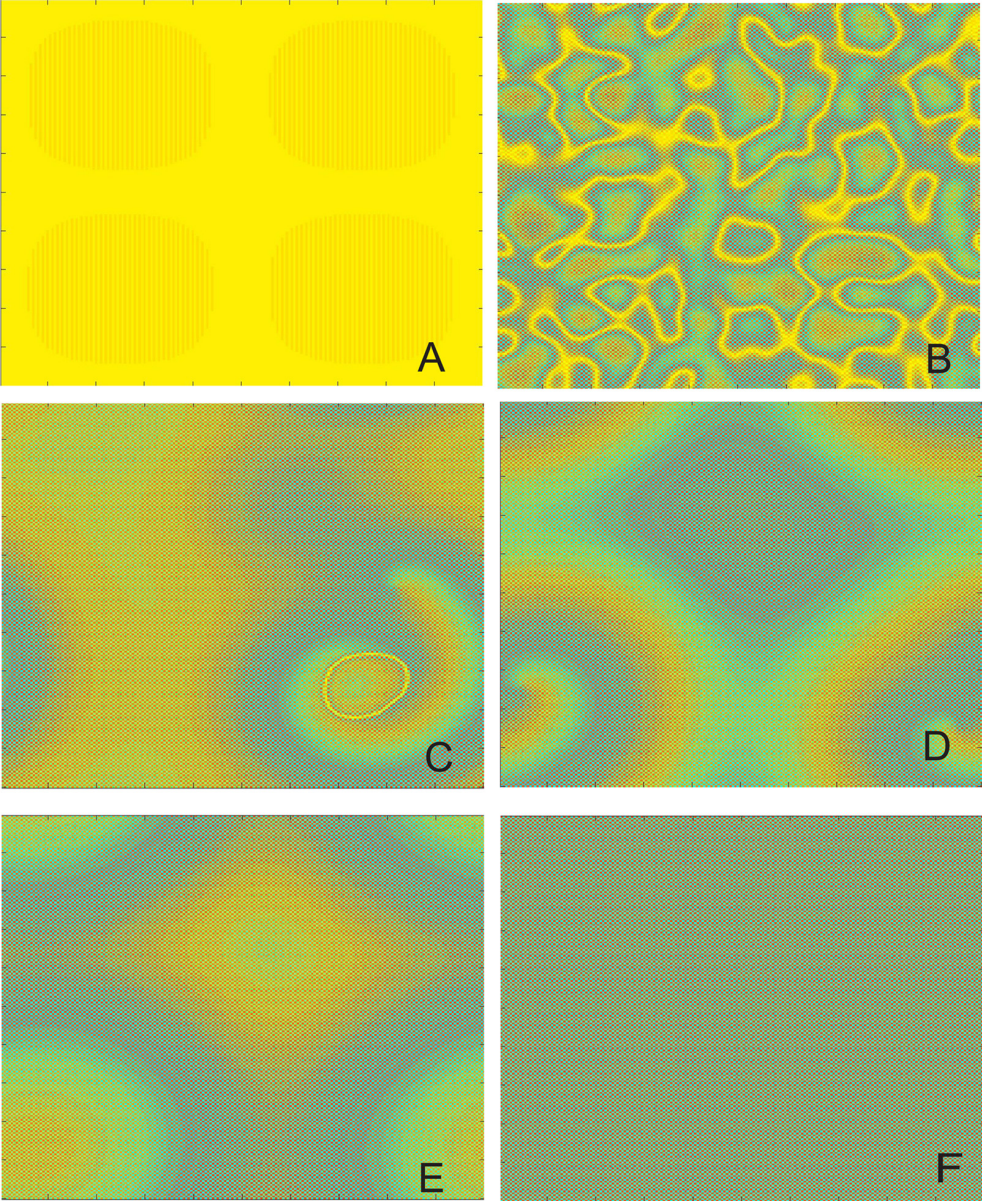
Spatial pattern of the time evolution at different instants. Snapshots of contour pictures of the time evolution of CML system at some instants with *λ* = 2.9 and *ε* = 0.34 in the Turing instability region. (A) *t* = 0. (B) *t* = 100. (C) *t* = 5000. (D) *t* = 10000. (E) *t* = 50000. (F) *t* = 100000.

To exhibit the difference between eigenvector initial value and random initial value, Fig 4A–4F exhibit in detail the distribution of time-evolutions for eigenvector initial value, In Fig 4B, the symmetry breaking around the fixed point can be observed. Fig 4C–4E show the self-organization process of the system, space-time periodic characteristics begin to appear, spiral wave patterns can be seen. Then with the evolution time proceeding, the spiral is tensility and broken down, steady periodic structures, namely, stationary wave emerge in Fig 4F.

Likewise, we performed lots of two-dimensional simulations with a system size of 201 × 201 space units. Contrast with Fig 2, similar patterns can also be realized dependent of eigenvector initial value.

## Conclusion

In this paper, we use a two-dimensional Logistic coupled map lattice to present mathematical mechanism of the effect of initial values on pattern development. Some asymptotic behavior between pattern formation and initial value determined by the corresponding eigenvectors of the eigenvalue for discrete Laplace operator can be found. Any initial value can be linearly expressed by corresponding eigenvectors, and patterns formation can be determined by selective the corresponding eigenvectors. We tested the effect by comparing the evolution of patterns with time starting from different initial values, and found that the patterns are sensitive to this factor. It has important consequences for modeling because it shows that quantitative prediction of the extent and control of patterns formation is possible when the initial values are well characterized.

## References

[1] Santos AM, Viana RL, Lopes S.R., de S. Pinto S.E. Batista A.M., Collective behavior in coupled chaotic map lattices with random perturbations. Physica A. 2008; 387: 1655–1668. 10.1016/j.physa.2007.09.051

[2] Xu L, Zhang G, Han B, Zhang L, Li MF, Han YT, Turing instability for a two-dimensional Logistic coupled map lattice. Phys. Lett. A. 2010; 374: 3447–3450. 10.1016/j.physleta.2010.06.065

[3] Sun GQ, Chakraborty A, Liu QX, Jin Z, Anderson KE, Li BL, Influence of time delay and nonlinear diffusion on herbivore outbreak. Commun Nonlinear Sci Numer Simulat 2014; 19: 1507–1518. 10.1016/j.cnsns.2013.09.016

[4] Sun GQ, Wu ZY, Wang Z, Jin Z, Influence of isolation degree of spatial patterns on persistence of populations.Nonlinear Dyn 2016; 83: 811–819. 10.1007/s11071-015-2369-6

[5] Murray JD, Mathematical Biology. Berlin: Springer; 1989 10.1007/978-3-662-08539-4

[6] Rangarajan G, Chen YH, Ding MZ, Generalized Turing patterns and their selective realization in spatiotemporal systems. Phys. Lett. A. 2003; 310: 415–422. 10.1016/S0375-9601(03)00447-X

[7] Almirantis Y, Pattern formation in a Turing’s type model with minimal reactional complexity. Comput Chem. 2004; 24: 159–170. 10.1016/S0097-8485(99)00057-1 10719635

[8] Field RJ, Burger M, Oscillations and travelling waves in chemical systems. John Wiley & Sons, New York, 1985.

[9] Enderlein J, Kuhnert L, Changing the Dynamical Behavior of Nonlinear Reaction Diffusion Systems by Stochastic Electric Fields. J. Phys. Chem. 1996; 100: 19642–19646. 10.1021/jp9615870

[10] Kytta K, Kaskia K, Barrioa RA, Complex turing patterns in non-linearly coupled systems. Physica A. 2007; 385: 105–114. 10.1016/j.physa.2007.06.034

[11] Li L, Jin Z, Li J, Periodic solutions in a herbivore-plant system with time delay and spatial diffusion. Appl Math Model. 2016; 40: 4765–4777. 10.1016/j.apm.2015.12.003

[12] Yusuke I, Hirofumi I, Takuya M, Turing instability in reaction–diffusion models on complex networks. Physica A. 2016; 457: 331–347. 10.1016/j.physa.2016.03.055

[13] Zhang YQ, Wang XY, Spatiotemporal chaos in mixed linear–nonlinear coupled logistic map lattice. Physica A. 2014; 402: 104–118. 10.1016/j.physa.2014.01.051

[14] Sun GQ, Zhang J, Song LP, Jin Z, Li BL, Pattern formation of spatial predator-prey system, Appl Math Comput. 2012; 218: 11151–11162.

[15] Sun GQ, Wang SL, Ren Q, Jin Z, Wu YP, Effects of time delay and space on herbivore dynamics: linking inducible defenses of plants to herbivore outbreak, Sci Rep. 2015; 5: 11246 10.1038/srep11246 26084812 PMC4471659

[16] Brovelli A, Malaguerra F, Barry DA, Bioclogging in porous media: model development and sensitivity to initial conditions. Environ. Modell. Softw. 2009; 24: 611–626. 10.1016/j.envsoft.2008.10.001

[17] Delcourt F, Taillandier P, Vidal F, Strehaiano P, Influence of pH, malic acid and glucose concentrations on malic acid consumption by Saccharomyces cerevisiae. Appl Microbiol Biotechnol. 1995; 43: 321–324. 10.1007/BF00172832 7612251

[18] Lagzi I, Izsak F, Stabilization and destabilization effects of the electric field on stochastic precipitate pattern formation. Chemical Physics. 2004; 303: 151–155. 10.1016/j.chemphys.2004.05.016

[19] White A, Begon M, Bowers RG, Host-pathogen systems in a spatially patchy environment. Proc. R. Soc. Lond. B. 1996; 263: 325–332. 10.1098/rspb.1996.0050 8920254

[20] Navickas Z, Smidtaite R, Ragulskis VA, The logistic map of matrices. Discrete Cont Dyn- B. 2011; 3: 927–944.

[21] Voroney JE, Lawniczak AT, Kapral R, Turing pattern formation in heterogenous media. Physica D. 1996; 99: 303–317. 10.1016/S0167-2789(96)00132-7

[22] Skudarnov PV, Lin CX, Wang MH, Pradeep N, Evolution of convection pattern during the solidification process of a binary mixture:effect of initial solutal concentration. Int. J. Heat Mass Transfer. 2002; 45: 5191–5200. 10.1016/S0017-9310(02)00224-7

[23] Nomura A, Miike A, Sakurai T, Numerical experiments on the Turing instability in the Oregonator model. J. Phys. Soc. Japan. 1997; 66: 598–606. 10.1143/JPSJ.66.598

